# Effect of surgical margin on postoperative prognosis in patients with solitary hepatocellular carcinoma: A propensity score matching analysis

**DOI:** 10.7150/jca.57896

**Published:** 2021-05-27

**Authors:** Zewen Zhou, Lunan Qi, Qiuyan Mo, Yingchun Liu, Xianguo Zhou, Zihan Zhou, Xiumei Liang, Shixiong Feng, Hongping Yu

**Affiliations:** 1 Guangxi Medical University Cancer Hospital, Nanning 530021, China.; 2 School of Public Health, Guangxi Medical University, Nanning 530021, China.; Zewen Zhou and Lunan Qi contributed equally to this work.

**Keywords:** surgical margin, postoperative prognosis, propensity score matching, hepatocellular carcinoma

## Abstract

**Objective:** The effect of surgical margin (SM) on the postoperative prognosis of patients with solitary hepatocellular carcinoma (HCC) remains controversial. This study aimed to evaluate the effect of SM on the postoperative prognosis of patients with solitary HCC by using propensity score matching (PSM).

**Methods:** Patients with solitary HCC who underwent liver resection were divided into a wide margin group (1.0 cm or more, group W) and a narrow margin group (< 1.0 cm, group N). Progression-free survival (PFS) and overall survival (OS) associated with the SM status and the factors influencing postoperative prognosis were evaluated.

**Results:** Before PSM, the indicators were not balanced between the two groups. PFS and OS were significantly lower in group N than group W. The factors affecting postoperative prognosis were international normalized ratio (INR), AST, capsule integrity, microvascular invasion, tumour embolus and tumour size. After PSM, data of both groups were balanced and comparable, and no significant differences in OS or PFS between the two groups. The INR in the above affecting factors was excluded.

**Conclusion:** For solitary HCC patients with negative SMs, SM size does not affect prognosis. INR, AST, capsule integrity, microvascular invasion, tumour embolus and tumour size are independent factors influencing the postoperative prognosis of solitary HCC patients.

## Introduction

A global statistical analysis of tumours in 2018 showed that the number of new-onset liver cancer (LC) cases was 841,080, accounting for 4.65% of new-onset cancer cases worldwide, and the number of LC-related deaths was 781,631, accounting for 8.18% of cancer deaths worldwide 1. The LC incidence and the number of LC cases differ greatly across the world 2. The LC incidence is the highest in East Asia, Southeast Asia, and sub-Saharan Africa and is low in Europe and America, while China has approximately half of the LC cases worldwide. Hepatitis B virus (HBV) infection is the leading cause of the high LC incidence in China 3. Primary LC includes hepatocellular carcinoma (HCC), intrahepatic cholangiocarcinoma, and combined hepatocellular carcinoma and cholangiocarcinoma 4. Clinically, HCC is the most common type, accounting for 85-90% of all LC cases.

Because LC usually has a high degree of malignancy, rapid disease progression, and tendentious recurrence and metastasis, it usually has a poor prognosis 5, 6. Surgery and liver transplantation are still the most important treatment methods for early-stage LC 7, 8. Many factors affect the postoperative prognosis of LC patients, such as tumour-node-metastasis (TNM) stage 7, Barcelona Clinic Liver Cancer (BCLC) stage 8, 9, albumin-bilirubin (ALBI) score 10, surgical margin (SM) 11-13, degree of tumour differentiation 14, the number of tumours 15, and tumour size 16. Some of these factors remain controversial. On the one hand, most of the evidence comes from retrospective studies, so the confounding factors have been difficult to control for; on the other hand, prospective studies often have relatively small sample sizes, which reduces the effectiveness of statistical tests.

In Chinese surgical practice, for LC surgery, the SM must be at least 1 cm away from the tumour. However, in practice, due to the special anatomical location of the tumour (e.g., the tumour is close to a large blood vessel), many patients still have a SM less than 1 cm. A series of studies have examined the effect of SM on the postoperative prognosis of HCC patients. A retrospective study on the postoperative prognosis of HCC patients in Japan showed that SM affected the prognosis of patients with tumour diameter ≤ 4 cm but had no effect on the prognosis of patients with tumour diameter > 4 cm 11. The effect of SM on the prognosis of LC patients was confirmed in another Japanese study, which showed that when tumour size was ≤ 2 cm, SM affected the prognosis of LC patients, but SM did not affect the prognosis of LC patients with tumour size > 2 cm 12. A study conducted in Taiwan also found a correlation between SM and the prognosis of LC patients 13. However, two other studies conducted in Taiwan showed that SM was not associated with postoperative prognosis in LC patients 17, 18.

Studies on the correlation between SM and the prognosis of the HCC population in China are still relatively rare. Considering the pathogenesis of LC and the ethnic specificity of China, it is worthwhile to explore the relationship between SM and prognosis in HCC patients in China. In this study, two groups of HCC patients were defined according to SM size, and the confounding factors in the two groups were controlled for by propensity score matching PSM to make the clinical data of the two SM groups balanced and comparable. The relationship between SM and the prognosis of HCC patients was further explored.

## Data and methods

### Study subjects

In a long-term follow-up cohort of HCC patients in the Guangxi Medical University Cancer Hospital, 817 patients who underwent liver resection after an initial diagnosis in our hospital from January 2013 to December 2015 were selected, and their clinical data and follow-up information were collected.

### Inclusion and exclusion criteria

The inclusion criteria were as follows: (1) HCC was confirmed by postoperative pathology; (2) liver function was Child-Pugh A or Child-Pugh B that could be improved to Child-Pugh A, and; (3) no adjuvant therapy was received before surgery. The exclusion criteria were as follows: (1) distant metastasis or advanced HCC; (2) severe heart, lung, kidney, or cerebrovascular diseases; and (3) positive SMs.

### Follow-up status

Start and end of follow-up: The surgery time was taken as the starting point of follow-up. The patients were followed up every 3 months for the first 3 years after surgery, then followed up every 6 months. For the patients who died due to HCC during the follow-up period, the death was the end point, and for the other patients, the end point was December 31, 2019. Postoperative recurrence was defined as two typical imaging findings, one imaging finding + elevated α-fetoprotein (AFP), or positivity on biopsy/resection. For patients with recurrence, treatments such as secondary surgery, radiofrequency ablation, transarterial chemoembolization, sorafenib, and best supportive care were recommended based on the recurrence pattern and functional liver reserve. Overall survival (OS) was defined as the time from first surgery to death from any cause or to the last follow-up for the patients lost to follow-up. Progression-free survival (PFS) was defined as the time from the first surgery to the earliest evidence of recurrence. Among the 817 patients, 78 were lost to follow-up, for a rate of loss to follow-up of 9.55%. If the clinical data of the patients who were lost follow-up were complete, and they were still included to ensure the authenticity of this study.

### Definition of SM

The SM refers to the shortest distance from the edge of the tumour to the resection line. In clinical practice, the SM is usually estimated by the naked eye of the surgeon and measured by a pathologist. For the surgeries included in this study, the surgical sections were consistent, and the SMs were determined by pathologists. The measurement criteria of SMs in the Department of Pathology were as follows: (1) the width of the resection margin was the distance from the tumour margin to the liver parenchyma; (2) for multinodular or satellite lesions, the tumour nodule closest to the edge was used as a reference; and (3) in the measurement of margin width for each dimension, the minimum value was defined as the narrowest width.

### Grouping

According to the SM size in the pathology report, the 817 patients were divided into a wide margin group (1.0 cm or more, group W, n = 325) and a narrow margin group (< 1.0 cm, group N, n = 492). Group W including 276 males and 49 females, with an average age of 49.27 ± 10.55 years. Group N including 407 males and 85 females, with an average age of 49.50 ± 11.22 years.

### PSM of indicators

This study matched the indicators from the following four categories: (1) baseline data: age, sex, and body mass index (BMI); (2) chronic diseases: liver cirrhosis, hypertension, and diabetes; (3) routine haematological and biochemical indicators: international normalized ratio (INR), aspartate aminotransferase (AST), alanine aminotransferase (ALT), serum a-fetoprotein (AFP), hepatitis B e-antigen (HBeAg), hepatitis B surface antigen (HBsAg), and HBV DNA; (4) tumour-related indicators: Edmondson grade, BCLC stage, ALBI score, platelet-to-lymphocyte ratio (PLR), neutrophil-to-lymphocyte ratio (NLR), capsule integrity, microvascular invasion, tumour embolus, and tumour size.

The basic information and underlying diseases of patients were obtained from the initial admission records. PLR is calculated as platelet count/lymphocyte count, and the optimal cut-off value obtained from the receiver operating characteristic (ROC) curve was used to divide the patients in a low PLR group and a high PLR group. NLR is calculated as neutrophil count/lymphocyte count, and the optimal cut-off value obtained from the ROC curve was used to divide the patients in a low NLR group and a high NLR group. ALBI score is calculated as -0.085 × [albumin (g/L)] + 0.66 × log_10_[bilirubin (mmol/L)] 19, see Table 1.

### Statistical analysis

SPSS (version 22.0; SPSS Inc., Chicago, IL, USA) was used for statistical analysis. Continuous variables are expressed as the mean ± standard deviation (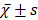), and the Z-test was used for comparison; the categorical data are expressed as the frequency, and the χ^2^ test was used for comparison. PSM was calculated using a logistic regression model, the one-to-one nearest-neighbour matching algorithm was used, the calliper width was 0.2, and there was no replacement. The balance between the variables of the two groups was evaluated through standardized differences.^20^ The survival analysis was performed using the Kaplan-Meier method, and survival was compared using the log-rank test. A Cox proportional hazard model was used for univariate and multivariate analyses, and potential risk factors (*P* < 0.05) in univariate analysis were input into the Cox regression model. When *P* < 0.05, the difference was considered statistically significant.

## Results

### Baseline data of the two groups before and after PSM

Before and after PSM, the results showed that the BCLC stage, tumour size, ALBI score, and AST were not balanced between the two groups (*P*<0.05). After PSM, there were 325 pairs included in the analysis, and all indicators of baseline data were comparable (*P*>0.05), see Table 2.

### Survival

**Figure 1:**
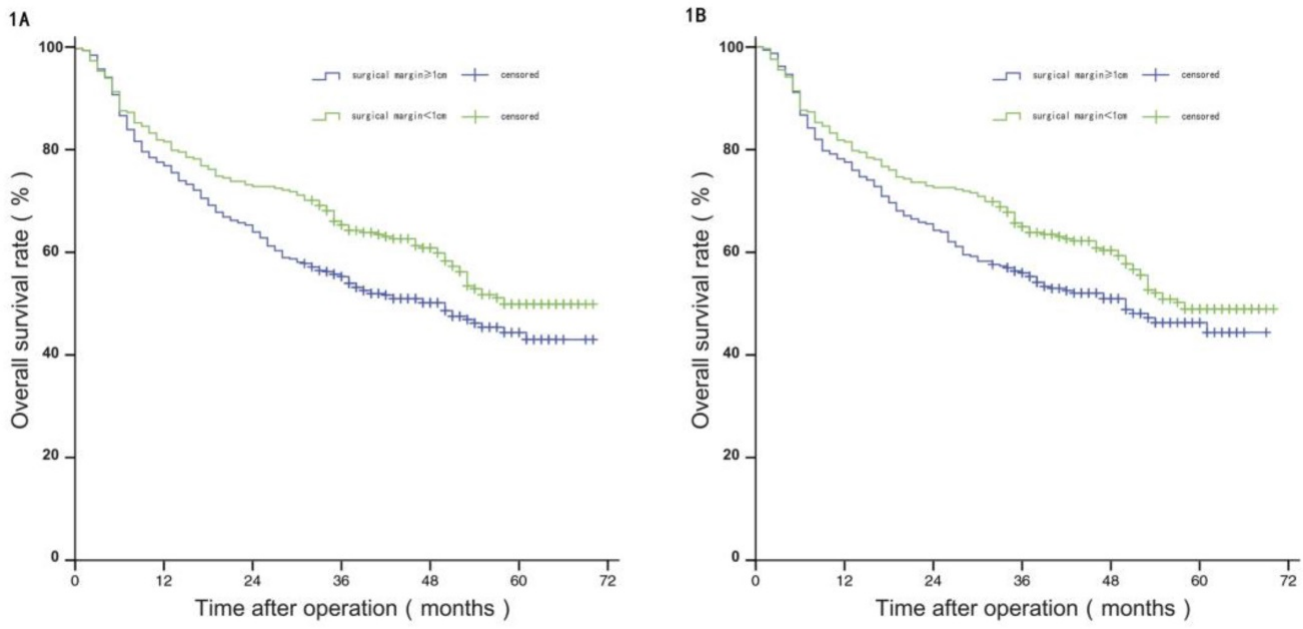
Comparison of OS between the two groups before and after propensity matching.

Before PSM, the median OS of patients in group W was 58 months, and the estimated OS rates at 1 year, 3 years, and 5 years were 81.50%, 65.20%, and 49.70%, respectively; and the median OS of patients in group N was 50 months, with estimated 1-year, 3-year, and 5-year OS of 75.80%, 55.30%, and 44.40%. The difference in OS between the two groups was statistically significant (*P*<0.05). After PSM, the median OS of patients in group W was 57 months, and the estimated OS rates at 1 year, 3 years, and 5 years were 81.20%, 64.60%, and 48.60%, respectively; and the median OS of patients in group N was 50 months, with estimated 1-year, 3-year, and 5-year OS of 77.20%, 55.60%, and 45.90%. The difference in OS between the two groups was not statistically significant (*P*>0.05), see Figure 1A and 1B.

**Figure 2:**
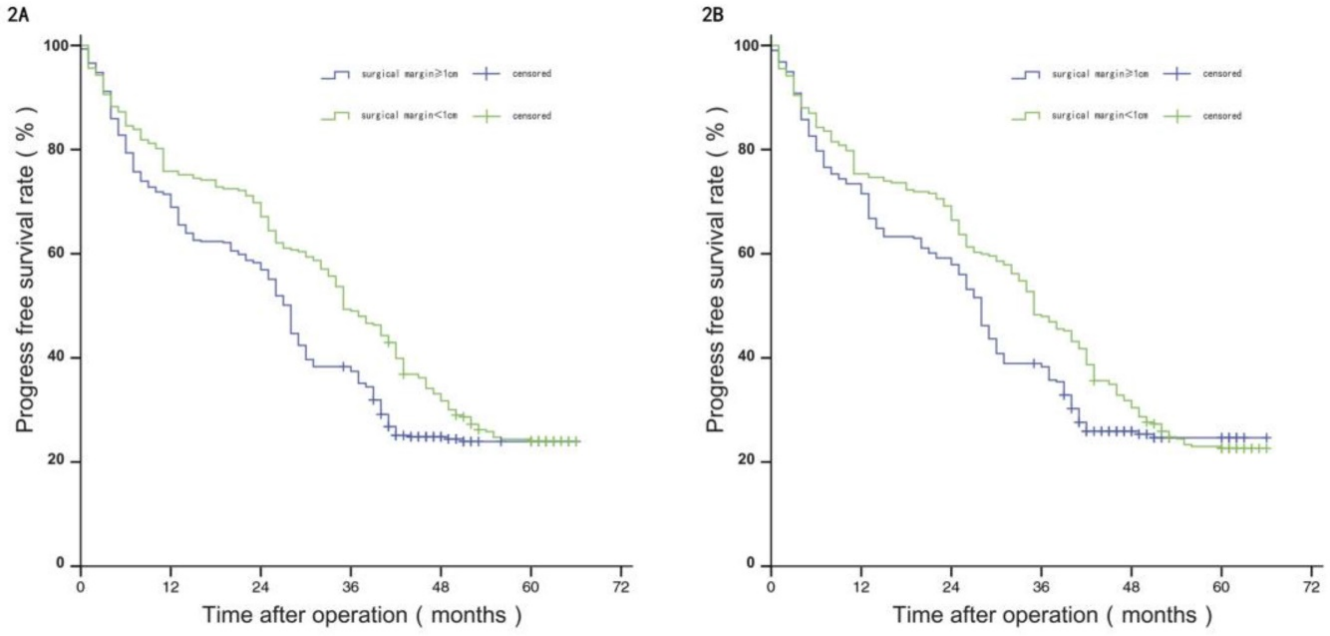
Comparison of RFS between the two groups before and after propensity matching.

Before PSM, the median PFS of patients in group W was 35 months, and the estimated PFS rates at 1 year, 3 years, and 5 years were 75.80%, 49.00%, and 24.00%, respectively; and the median PFS of patients in group N was 28 months, with estimated 1-year, 3-year, and 5-year PFS of 68.90%, 37.40%, and 23.90%. The difference in PFS between the two groups was statistically significant (*P*<0.05). After PSM, the median PFS of patients in group W was 35 months, and the estimated PFS rates at 1 year, 3 years, and 5 years were 75.30%, 47.90%, and 22.60%, respectively; and the median PFS of patients in group N was 28 months, with estimated 1-year, 3-year, and 5-year PFS of 71.50%, 38.30%, and 24.70%. The difference in PFS between the two groups was not statistically significant (*P*>0.05), see Figure 2A and 2B.

### Factors influencing postoperative survival

Before PSM, albumin, INR, AST, ALT, AFP, HBV DNA, Edmondson grade, BCLC stage, ALBI score, PLR, NLR, capsule integrity, microvascular invasion, tumour embolus, tumour size, and SM were the possible influencing factors in the univariate analysis (*P*<0.05). After multivariate analysis, INR, AST, capsule integrity, microvascular invasion, tumour embolus, and tumour size were kept as independent factors of the postoperative survival of patients (*P*<0.05), see Table 3.

After PSM, albumin, INR, AST, AFP, HBV DNA, Edmondson grade, BCLC stage, ALBI score, PLR, NLR, capsule integrity, microvascular invasion, tumour embolus, and tumour size were identified as possible influencing factors in the univariate analysis (*P*<0.05). In the multivariate analysis, AST, capsule integrity, microvascular invasion, tumour embolus, and tumour size were kept as independent factors influencing the postoperative survival of patients (*P*<0.05), see Table 4.

## Discussion

In a cohort study, PSM provides researchers with the ability to balance all the hypothesized risk factors between groups and makes it easy to check whether covariates are balanced 20. PSM can reduce bias and improve the effectiveness of estimates by excluding unmatched study subjects 21, 22. Whether the SM is an independent factor affecting the postoperative prognosis of HCC patients remains controversial. To exclude the interference of confounding factors and explore the effect of SM on the postoperative survival of HCC patients, this study used PSM to match the indicators of four categories, i.e., basic information, underlying diseases, tumour-related indicators, and routine haematological and biochemical indicators. In addition to the common indicators from previous studies, BMI, hypertension, and diabetes were also included. In clinical practice, a growing number of LC patients have abnormal BMI or underlying diseases, such as hypertension and diabetes. Abnormal BMI 23, diabetes 24, 25 and hypertension 26 are related to the incidence of LC; however, studies on the relationship between these three factors and the prognosis of HCC are still rare, so it is of certain practical significance to explore the relationship between these factors and the prognosis of HCC.

Our results showed that before PSM, SM had an impact on the survival of the patients. The PFS and OS of the patients in the W group (SM ≥ 1 cm) were longer than those in the N group (SM < 1 cm). After PSM, the differences in PFS and OS of the two groups were not significantly different. The results of this study, to some extent, explain the controversial conclusions from different studies in Taiwan 13, 17, 18. Due to the presence of confounding factors, the baseline data of patients in two different groups may not be balanced, and such biases lead to different results in different studies.

The results of this study are different from the results of similar studies by Japanese scholars 11, 12. Of the two studies in Japan on the effect of SM on the surgical prognosis of LC patients, one used tumour size ≤ 2 cm as the cut-off value, and the other used tumour size ≤ 4 cm. One study showed that when the tumour size was greater than the cut-off value (2 cm), the SM did not affect the prognosis of patients 11. However, the other study showed when the tumour size exceeds 4 cm, 10 mm of TW was inadequate to achieve curability and was linked to a recurrence 12. Tumour size is a possible confounding factor, and this study also included tumour size as an indicator in PSM. However, the incidence of HCC in China is mostly related to HBV infection 3, while the incidence of HCC in Japan is closely related to hepatitis C virus (HCV) 27. Japan emphasizes early screening for tumours, so the tumour size is usually smaller than that in China. Based on the margin-size characteristics of LC patients in south China, 5 cm was used as the cut-off value in this study.

Our results also differ from the results of a meta-analysis on the influence of SM on prognosis of patients 28. That meta-analysis concluded that SM was related to the prognosis of patients. However, its seven included papers mostly lacked any description of whether the SM was negative. Five of them had small samples, and two papers did not give the follow-up time.

After confirming that there was no correlation between the SM and the prognosis of HCC patients, we further analysed the factors influencing the prognosis of HCC patients. To compare a real-world analysis and a cohort analysis on the factors affecting the postoperative prognosis of HCC patients, univariate and multivariate analyses were performed on the factors influencing the postoperative prognosis of HCC patients before and after PSM. The results showed that in the real-world analysis, the independent factors influencing the postoperative prognosis of patients with solitary HCC were INR, AST, capsule integrity, microvascular invasion, tumour embolus, and tumour size. After PSM, INR was excluded, but the other factors were retained. This result suggests that in a real-world study and a cohort study, the prognostic factors for patients with solitary HCC are different. The reason may be that the inclusion and screening criteria of cohort studies exclude some patients, so the prognostic factors of these patients are also excluded.

BCLC stage 8, 9, ALBI score 10, and tumor differentiation 14 are generally considered independent factors affecting the postoperative prognosis of HCC patients. In our univariate analysis, there was also a statistically significant association between these factors and prognosis, but all three factors were ultimately excluded from the multivariate model. Edmondson grade was reported to predict the survival of patients with primary clear cell carcinoma of liver after curative resection 29, which was also a crucial predictor of survival in HCC without microvascular invasion 30. Whether BCLC stage, ALBI score, and degree of tumour differentiation affect the postoperative prognosis of HCC patients requires more studies to confirm. AFP was also statistically significant in univariate analysis, but negatively in multivariate analysis. It is still controversial whether preoperative AFP level acts as an independent prognostic factor in patients undergoing resection for HCC 31,32.

Albumin has often been used as a factor in some scores or ratios to explore its relationship with the postoperative prognosis of HCC patients, such as the platelet-albumin-bilirubin score 33, ALBI score 34, and albumin-to-alkaline phosphatase ratio 35. In this study, albumin was associated with prognosis in univariate analysis but was not included in the multivariate model. In the study by Wang et al. 36, PLR and NLR influenced the postoperative prognosis of HCC patients, and both were excluded from the final model in this study. In addition, our results showed that BMI, hypertension, and diabetes were unrelated to the postoperative prognosis of HCC patients.

Both INR and AST are measured in blood routine examination. INR can reflect the coagulation function of patients, and AST can reflect the degree of hepatic parenchymal damage. INR can be used as a predictor of disease severity in patients with colorectal cancer, but studies on the relationship between INR and postoperative prognosis of HCC patients are rare. The relationship between INR and the prognosis of HCC should be validated in studies with a large HCC sample size. The effect of AST level on the postoperative prognosis of HCC patients has also been confirmed in other studies. One study showed that patients with high AST had a short OS, which was consistent with the conclusions of this study 37. The relationships between the prognosis of HCC patients and tumour capsule integrity 38, 39, microvascular invasion 40-42, and tumour embolus 43, 44 have been confirmed in many studies, and this study again confirmed the validity of these independent influencing factors in a LC population in southern China. The relationship between tumour size and the postoperative prognosis of HCC patients remains controversial. Some studies suggested that tumour size was associated with the prognosis of patients 16, which is consistent with the conclusion of this study, but other study showed that tumour size and prognosis were not correlated 45.

This study had a large sample size and controlled for confounding factors between groups through PSM, so the conclusions obtained were reliable. However, this study still had limitations because it was a single-centre study targeting HCC patients in southern China. Among patients with negative SMs, the SM did not affect the prognosis, and NLR, AST, capsule integrity, microvascular invasion, tumour embolus, and tumour size were independent influencing factors of the postoperative prognosis of HCC patients.

## Figures and Tables

**Table 1:** Assignment table of research indexes

Index	Assignment rules
**Baseline data**	
age	<60 year=1, ≥60 year=2
sex	male=1, female=2
BMI	18.5 kg/m^2^ ~ 23.9 kg/m^2^ =1, others=2
**Chronic diseases**	
liver cirrhosis	no=0, yes=1
hypertension	no=0, yes=1
diabetes	no=0, yes=1
**Routine haematological and biochemical indicators**	
albumin	35 ~ 51 g/L=1, others=2
total bilirubin	3.4 ~ 17.1 μmol/L=1, others=2
INR	0.8 ~ 1.2=1, others=2
AST	≤40 U/L=1, >40 U/L=2
ALT	≤40 U/L=1, >40 U/L=2
AFP	<400 μg/L=1, ≥400 µg/L=2
HBeAg	negative=0, positive=1
HBsAg	negative=0, positive=1
HBV DNA	negative=0, positive=1
**Tumour-related indicators**	
Edmondson grade	III-IV =1, I ~ II =2
BCLC stage	0 =1, A =2, B =3, C =4
ALBI score	< -2.60=1, -2.60 ~ -1.39=2, > -1.39=3
PLR (before the matching)	≤199.48=1, >199.48=2
PLR (after the matching)	≤197.74=1, >197.74=2
NLR (before the matching)	≤2.34=1, >2.34=2
NLR (after the matching)	≤2.26=1, >2.26=2
capsule integrity	integrity =0, incomplete or without =1
microvascular invasion	no=0, yes=1
tumour embolus	no=0, yes=1
tumour size	< 5 cm=1, ≥ 5 cm=2

**Table 2:** Baseline data of the two groups

Index	Before PSM			After PSM		
	Group W (n=325)	Group N (n=492)	*P*	Group W (n=325)	Group N (n=325)	*P*
**Age**			0.406			0.396
<60 years	276	407		276	268	
≥60 years	49	85		49	57	
Male	274	421	0.621	274	277	0.743
Abnormal BMI	131	181	0.311	131	120	0.376
Liver cirrhosis	145	215	0.796	145	143	0.875
Hypertension	33	36	0.154	33	23	0.162
Diabetes	39	41	0.084	39	26	0.089
Abnormal albumin	49	74	0.944	49	46	0.739
Abnormal total bilirubin	60	110	0.192	60	65	0.619
Abnormal INR	36	72	0.142	36	43	0.401
AST>40 U/L	123	221	0.045	123	140	0.174
ALT>40 U/L	109	186	0.214	109	120	0.366
AFP≥400 µg/L	135	230	0.143	135	155	0.115
HBeAg (+)	78	137	0.222	78	89	0.323
HBsAg (+)	288	425	0.349	288	277	0.201
HBV DNA	204	317	0.629	204	210	0.625
**Edmondson grade**			0.788			0.633
I-II	192	286		192	186	
III-IV	133	206		133	139	
**BCLC stage**			0.023			0.211
0	11	18		11	13	
A	179	217		179	153	
B	46	87		46	49	
C	89	170		89	110	
**ALBI score**			0.020			0.082
< -2.60	98	106		98	73	
-2.60 ~ -1.39	221	374		221	246	
> -1.39	6	12		6	6	
**PLR**			0.503			0.507
≤199.48	274	406		274	280	
>199.48	51	86		51	45	
**NLR**			0.035			0.156
≤2.34	198	263		188	170	
>2.34	127	229		137	155	
Incomplete or without capsule	116	194	0.281	116	121	0.684
Microvascular invasion	153	206	0.142	153	145	0.529
Tumour embolus	239	332	0.065	239	220	0.102
Tumour size ≥ 5 cm	202	343	0.025	202	219	0.163

**Table 3:** Univariate and multivariate survival analysis before PSM

Index	Univariate analysis			Multivariate analysis		
	*P*	*HR*	95%CI	*P*	*HR*	95%CI
age	0.663					
sex	0.137					
BMI	0.136					
liver cirrhosis	0.551					
hypertension	0.744					
diabetes	0.165					
albumin	0.013	1.424	1.079-1.880	0.429		
total bilirubin	0.895					
INR	0.019	1.422	1.060-1.907	0.049	1.364	1.001-1.859
AST	<0.001	2.085	1.681-2.586	0.028	1.339	1.033-1.736
ALT	0.019	1.300	1.044-1.618	0.785		
AFP	<0.001	1.573	1.270-1.948	0.228		
HBeAg	0.102					
HBsAg	0.369					
HBV DNA	0.009	1.362	1.079-1.719	0.456		
Edmondson grade	<0.001	0.688	0.555-0.852	0.229		
BCLC stage	<0.001	2.052	1.820-2.313	0.149		
ALBI score	0.007	1.394	1.095-1.775	0.623		
PLR	<0.001	1.931	1.500-2.485	0.669		
NLR	<0.001	1.784	1.439-2.211	0.396		
capsule integrity	<0.001	2.408	1.943-2.985	<0.001	1.861	1.486-2.330
microvascular invasion	<0.001	2.103	1.671-2.646	0.019	1.345	1.050-1.724
tumour embolus	<0.001	3.592	2.895-4.458	0.015	1.680	1.104-2.557
tumour size	<0.001	2.965	2.246-3.915	0.004	1.603	1.162-2.211
surgical margin	0.042	0.792	0.633-0.992	0.095		

**Table 4:** Univariate and multivariate survival analysis after PSM

Index	Univariate analysis			Multivariate analysis		
	*P*	*HR*	95%CI	*P*	*HR*	95%CI
age	0.574					
sex	0.131					
BMI	0.305					
liver cirrhosis	0.413					
hypertension	0.731					
diabetes	0.653					
albumin	0.189					
total bilirubin	0.426					
INR	0.019	1.422	1.060-1.907	0.408		
AST	<0.001	2.195	1.728-2.788	0.013	1.394	1.071-1.814
ALT	0.153					
AFP	<0.001	1.573	1.240-1.995	0.317		
HBeAg	0.311					
HBsAg	0.177					
HBV DNA	0.033	1.322	1.023-1.708	0.731		
Edmondson grade	0.004	0.704	0.555-0.893	0.693		
BCLC stage	<0.001	2.071	1.818-2.360	0.191		
ALBI score	0.021	1.359	1.046-1.764	0.984		
PLR	<0.001	1.097	1.002-1.204	0.229		
NLR	<0.001	1.772	1.396-2.251	0.614		
capsule integrity	<0.001	2.294	1.807-2.911	<0.001	1.752	1.366-2.247
microvascular invasion	<0.001	2.103	1.671-2.646	0.031	1.344	1.028-1.759
tumour embolus	<0.001	3.768	2.963-4.790	0.028	1.709	1.058-2.760
tumour size	<0.001	3.068	2.256-4.172	0.018	1.541	1.076-2.207
surgical margin	0.067					
